# Exploiting the Transcriptome of Euphrates Poplar, *Populus euphratica* (Salicaceae) to Develop and Characterize New EST-SSR Markers and Construct an EST-SSR Database

**DOI:** 10.1371/journal.pone.0061337

**Published:** 2013-04-11

**Authors:** Fang K. Du, Fang Xu, Hong Qu, Sisi Feng, Jijun Tang, Rongling Wu

**Affiliations:** 1 Center for Computational Biology, National Engineering Laboratory for Tree Breeding, College of Biological Sciences and Biotechnology, Beijing Forestry University, Beijing, China; 2 Center for Bioinformatics, National Laboratory of Protein Engineering and Plant Genetic Engineering, College of Life Sciences, Peking University, Beijing, China; 3 Department of Computer Science and Engineering, University of South Carolina, Columbia, South Carolina, United States of America; CIRAD, France

## Abstract

**Background:**

Microsatellite markers or Simple Sequence Repeats (SSRs) are the most popular markers in population/conservation genetics. However, the development of novel microsatellite markers has been impeded by high costs, a lack of available sequence data and technical difficulties. New species-specific microsatellite markers were required to investigate the evolutionary history of the Euphratica tree, Populus euphratica, the only tree species found in the desert regions of Western China and adjacent Central Asian countries.

**Methodology/Principal Findings:**

A total of 94,090 non-redundant Expressed Sequence Tags (ESTs) from P. euphratica comprising around 63 Mb of sequence data were searched for SSRs. 4,202 SSRs were found in 3,839 ESTs, with 311 ESTs containing multiple SSRs. The most common motif types were trinucleotides (37%) and hexanucleotides (33%) repeats. We developed primer pairs for all of the identified EST-SSRs (eSSRs) and selected 673 of these pairs at random for further validation. 575 pairs (85%) gave successful amplification, of which, 464 (80.7%) were polymorphic in six to 24 individuals from natural populations across Northern China. We also tested the transferability of the polymorphic eSSRs to nine other Populus species. In addition, to facilitate the use of these new eSSR markers by other researchers, we mapped them onto Populus trichocarpa scaffolds in silico and compiled our data into a web-based database (http://202.205.131.253:8080/poplar/resources/static_page/index.html).

**Conclusions:**

The large set of validated eSSRs identified in this work will have many potential applications in studies on P. euphratica and other poplar species, in fields such as population genetics, comparative genomics, linkage mapping, QTL, and marker-assisted breeding. Their use will be facilitated by their incorporation into a user-friendly web-based database.

## Introduction

*Populus euphratica* Oliv. (Salicaceae) inhabits semi-arid areas, with a natural distribution ranging from Western China and the Middle East to Spain and Western Morocco. As a desert tree, it is considered to be salinity and drought tolerant [1]. Moreover, it plays an important role in maintaining the ecological equilibrium of desert environments by counteracting the effects of dust storms, stabilizing sand, and retaining water [2]. Changes in its habitats and distribution have caused natural stands of *P. euphratica* to diminish over the last few decades, and some populations have disappeared [3]. *P. euphratica* is a useful model organism for studying salt and drought resistance mechanisms in trees because it can tolerate NaCl concentrations of up to 450 mM [4], [5] and mannitol concentrations up to 400 mM [6] in controlled experiments. Much of the work conducted on this species to date has focused on the physiological mechanisms responsible for its resistance to abiotic factors (e.g. [7] and references therein). More recent studies using Amplified Fragment Length Polymorphism (AFLP) or microsatellite analysis based on polyacrylamide gel electrophoresis (PAGE) with silver staining have provided useful information on its genetics and evolutionary history [8–0]. However, partly because of the scarcity of suitable molecular markers, much remains to be learned about the genetic factors responsible for the ability of *P. euphratica* to cope with various adverse environmental conditions.

SSRs (simple sequence repeats) [11] are tandem repeated DNA sequences with a repeating motif of one to six base pairs. They are also known as STRs (short tandem repeats) [12], SSLPs (simple sequence length polymorphisms) [13], and microsatellites [14]. SSRs are common and widely distributed in prokaryotic and eukaryotic genomes [15]. Because of their high rates of mutation, they tend to be highly polymorphic [16]. This makes it possible to distinguish between alleles based on their length using either classical PAGE, or by DNA sequencing when working on a larger scale or when higher accuracy is required [17]. Because of these useful properties, SSRs are the most popular markers in population genetics [17] and have been used in many applications such as gene tagging, Quantitative Trait Locus (QTL) mapping, marker assisted selection (MAS), parentage analysis, fingerprinting, and phylogenetic and taxonomic studies [18]. Two categories of SSRs can be defined based on their locations in the genome: Expressed Sequence Tag-SSRs (eSSRs) are embedded in transcribed sequences [19], while genomic SSRs (gSSRs) are embedded in both transcribed and non-transcribed sequences. Until recently, the lack of the detailed genomic information for many plants has meant that SSR marker development was primarily conducted using selective hybridization strategies, which require fragmented genomic DNA that is bound to either a nylon membrane using repeat-containing probes or to biotinylated probes that are subsequently captured on streptavidin-coated beads [20]–[23]. This is complicated by the difficulty of preparing uniformly digested genome DNA, the cumbersome nature of the molecular cloning, the limited scope for obtaining sufficiently long sequences, and the high cost of the overall process. However, thousands of ESTs can be obtained from a single cDNA library comprising fragments with lengths of 300–500 bp, from which plenty of eSSRs can be designed. Because ESTs are conserved across phylogenetically related species, eSSRs developed for one species are often highly transferable and this characteristic has made them the markers of choice in comparative mapping and QTL analysis [24]. In recent years, traditional Sanger sequencing and second generation sequencing methods have produced huge numbers of EST sequences that have been archived in public databases. These databases offer a great opportunity for identifying SSRs through data mining, especially for non-model organisms for which few genomic resources and reference sequences are available. ESTs have been used to design SSR markers for many plant taxa, including crops (rice [25], maize [26], wheat [26], [27], cotton [28], etc) and trees (coffee [29], conifers [24], [30], rubber tree [31], oak [24] and poplar [32], etc.).

Despite its ecological importance, there have been comparatively few genetic studies focusing on *P. euphratica*. In particular, there is a lack of genomic studies using methods such as QTL analysis, for which a relatively large battery of markers is required. To our knowledge, only 12 gSSRs have been reported for *P. euphratica* to date [33]. The development of SSR markers in this species has been hindered by a lack of genomic data as well as the expensive and laborious nature of the methods used to develop SSRs from genomic libraries.

The study reported herein was conducted to address these issues by: (1) screening *P. euphratica* ESTs to identify new SSRs, (2) developing new eSSR markers and mapping them onto *P. trichocarpa* scaffolds *in silico*, (3) screening the newly-identified polymorphic eSSR markers, (4) validating the transferability of the new polymorphic eSSRs to other related species, and (5) compiling all of the new results in a freely-accessible web-based database.

## Results

### Frequency and distribution of eSSRs

Our ESTs originated from two resources: the National Center for Biotechnology Information (NCBI) database (http://www.ncbi.nlm.nih.gov/nucest/) and a published paper reporting *P. euphratica* transcriptome data [34]. We initially obtained 13,979 ESTs from the NCBI, most of which were originally identified by studying a cDNA library derived from salt-stressed leaves of two-year-old seedlings. We also included 94,196 UniGenes from a publication describing the first deep transcriptomic analysis of living tissues from desert-grown trees and from two types of calluses (salt-stressed and unstressed) using next generation sequencing and transcriptome analysis [34].

This starting dataset of 108,175 ESTs was reduced by removing 266 sequences: 109 that were less than 100 bp in length, 148 that were similar to chloroplast genes, 3 that were similar to mitochondrial genes, and 6 that were similar to vector sequences. We then clustered 107,909 valid ESTs using the CD-HIT [35] with a 90% similarity's cut-off, which left a total of 94,090 unique ESTs with a combined length of 62,650,653 bp sequences identified.

**Figure 1:**
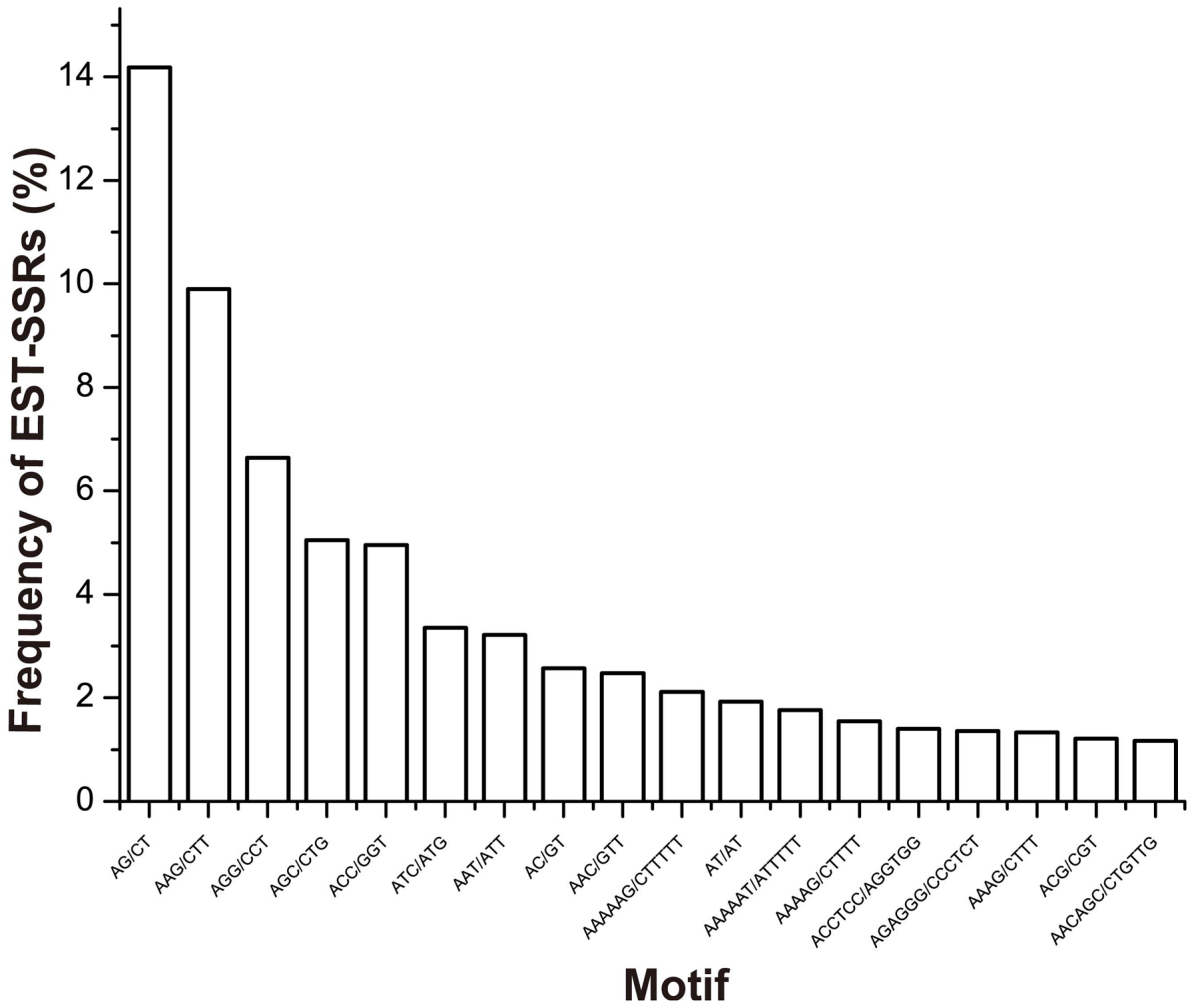
Frequency of different motifs among the eSSRs of *P. euphratica*.

The perl script MIcroSAtellite identification tool (MISA) was used to search for SSRs within the set of 94,090 ESTs [36] (http://pgrc.ipk-gatersleben.de/misa). A total of 4,202 SSRs were identified in 3,839 ESTs (4% of the entire EST data set), with 3,528 ESTs containing only one SSR and 311 containing multiple SSRs (Table S1). On average, every 15 kb of the *P. euphratica* transcripts contained one SSR. The most abundant SSR type among the 4,202 identified were trinucleotides (37%), followed by hexanucleotides (33%), dinucleotides (19%), pentanucleotides (6%), and tetranucleotides (4%; Figure S1 and Table S2). 175 compound SSRs were identified, representing around 4% of the 4,202 SSR loci. Overall, the average number of SSR repeats across all motifs was six. The dominant motif among dinucleotides SSRs was AG/CT (14% of all SSR motifs), followed by AC/GT (3%) and AT/TA (2%). The most common trinucleotides motif was AAG/CTT (10%), followed by AGG/CCT (7%) and AGC/GCT (5%). AAAN, AAAAN and AAAAAN were most common tetra-, penta-, and hexanucleotides SSR motifs respectively (Figure 1).

**Figure 2:**
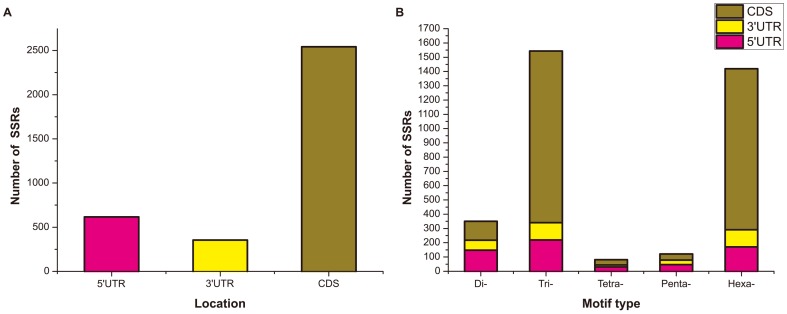
Distribution of eSSRs across the 5′ UTR, 3′ UTR and CDS in *P. euphratica*. (A). Abundance of eSSRs with different motif sizes and their distributions in non-coding and coding region (B).

We also investigated the eSSRs' locations by mapping the eSSR regions on the genome of *P. trichocarpa* [33], the first tree to have its whole genome sequenced. In total, 3,152 (75%) of the identified *P. euphratica* eSSRs could be located in the *P. trichocarpa* genome. Most (72%) of them were located in coding regions, with only 18% being located in 5′-untranslated regions (5′-UTR) and 10% in 3′-untranslated regions (3′-UTR) (Figure 2A). Of the SSRs identified in coding regions, 47% were trinucleotides and 44% were hexanucleotides. Of the eSSRs identified in non-coding regions, 35% were trinucleotides and 30% were hexanucleotides, with most of them being embedded in 5′ UTRs (63%) (Figure 2B).

### Marker development

We were able to design primers for 3,069 (73%) of the 4,202 eSSRs that were identified. In most cases where it was not possible to design primers for a given eSSR, the failure was because its flanking sequences were too short. Primers were designed using a two-step process. First, for each eSSR, we selected the three primer pairs that achieved the best scores according to the design criteria of the software used. Data on the primers, including the sequences of the forward/reverse primers as well as their melting temperatures (Tm), expected amplicon size, corresponding EST ID, type of SSR, amplification status, and the degree of polymorphism of the target locus are provided in the supplemental data (Table S3). Second, we selected 673 primer pairs at random (one pair for each eSSR) for 413 di-, 154 tri-, one tetra-, 15 penta- and 90 hexanucleotides SSRs to perform further validation. Amplicons were successfully generated using 575 (85%) of the tested primer pairs.

### Polymorphism and the genetic diversity of eSSRs in *P. euphratica*

To evaluate the polymorphism of the identified eSSR markers, we performed experiments using the 575 primer pairs that successfully generated amplicons. The 575 SSR loci targeted by these primers comprise 315 dinucleotides, 154 trinucleotides, one tetranucleotides, 15 pentanucleotides, and 90 hexanucleotides repeats. We used these primer pairs to amplify six, 12 or 24 DNA samples of *P. euphratica* individuals that were randomly selected from a natural population in Xinjiang Province, Northwestern China. 464 (80.7%) of the amplified eSSRs showed some degree of polymorphism, with Polymorphism Information Content (PIC) [37] values ranging from 0.062 to 0.895, allele numbers (*N*_A_) ranging from two to 13, and expected heterozygosity (*H*_E_) values ranging from 0.067 to 0.942 (Table S4). Of the polymorphic eSSRs, 260 (56%) were dinucleotides eSSRs, 130 (28%) were trinucleotides eSSRs, 59 (13%) were hexanucleotides eSSRs, 14 (3%) were pentanucleotides eSSRs and one was a tetranucleotides.

### Cross species transferability of eSSRs in *Populus*

**Figure 3:**
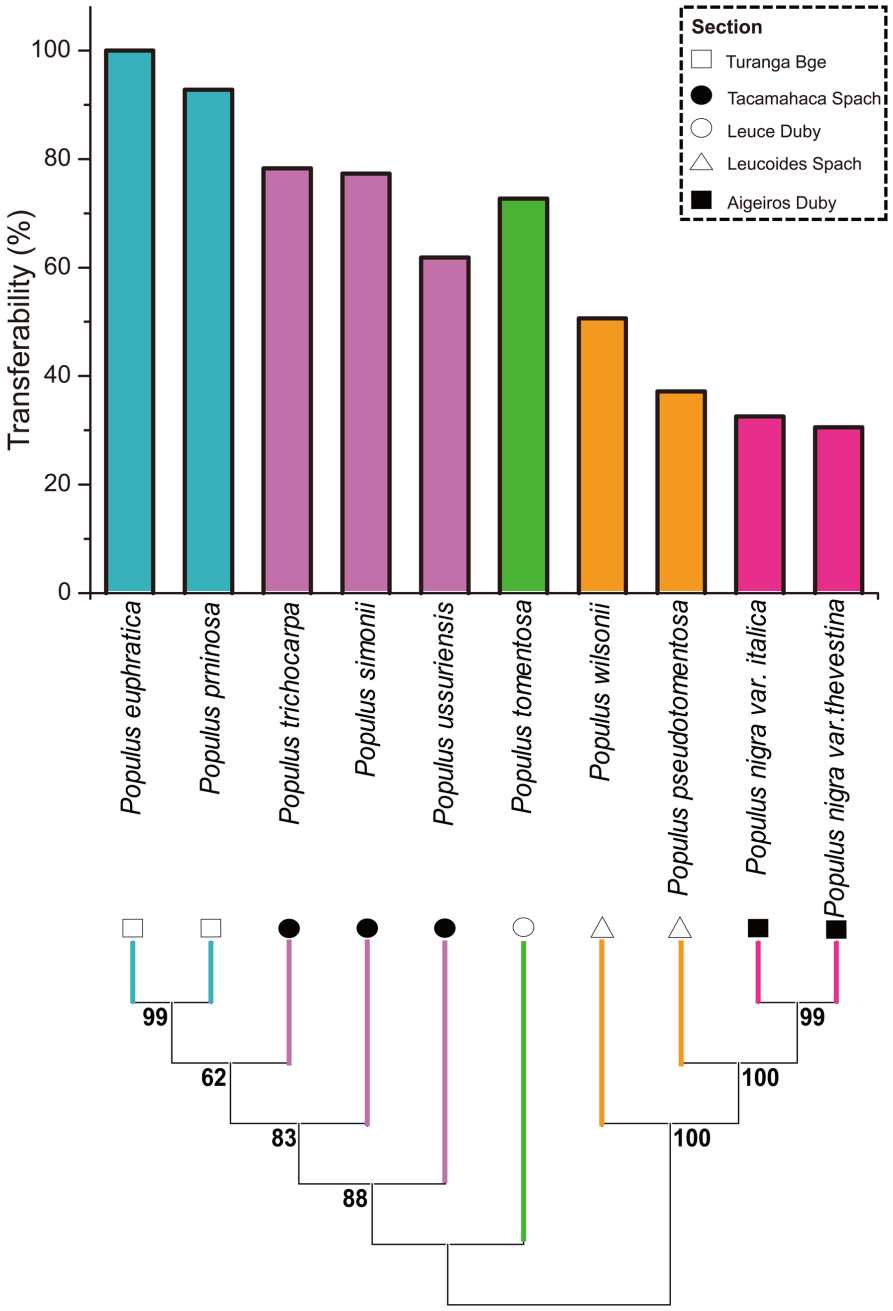
Transferability of polymorphic eSSRs. The uppermost figure shows the transferability of the 305 polymorphic eSSRs over nine species of *Populus* and *P. euphratica*. The lower figure shows a Neighbor-Joining tree for the tested species based on eSSR transferability. The box in the upper right-hand side of the figure shows the symbols used to indicate species belonging to different sections of the genus *Populus*.

In order to generate transferable eSSR markers for genetic studies on other *Populus* species, a total of 302 polymorphic primer pairs developed for *P. euphratica* were used to amplify DNA samples from nine other *Populus* species belonging to five sections (Aigeiros, Leucoides, Populus, Tacamahaca, Turanga) of the genus. The tested eSSRs exhibited high levels of transferability in *P. pruinosa* (>90%), *P. ussuriensis* (78%), *P. trichocarpa* (77%) and *P. tomentosa* (73%). Moderate levels of transferability were observed in *P. simonii* and *P. wilsonii* (62% and 51%, respectively; Figure 3 and Table S5). However, the levels of transferability for *P. pseudoglauca*, *P. nigra* var. *thevestina* and *P. nigra* var. *italica* were rather low (37%, 33% and 31% respectively).

To determine whether these transferability values might reflect the phylogenetic relationships between species in the *Populus* genus, we constructed a Neighbor joining tree using transferability data for 302 primers (Figure 3). Inspection of the tree revealed two groups of phylogenetically closely related species: *P. pruinosa and P. euphratica* in section Turanga Bge, and *P. nigra* var. *thevestina* and *P. nigra* var. *italica* in section Aigeiros Duby. However, no relationships involving species in the other three sections of the genus were identified.

### *In silico* mapping of eSSRs onto the *P. trichocarpa* genome and their functional annotation

**Figure 4:**
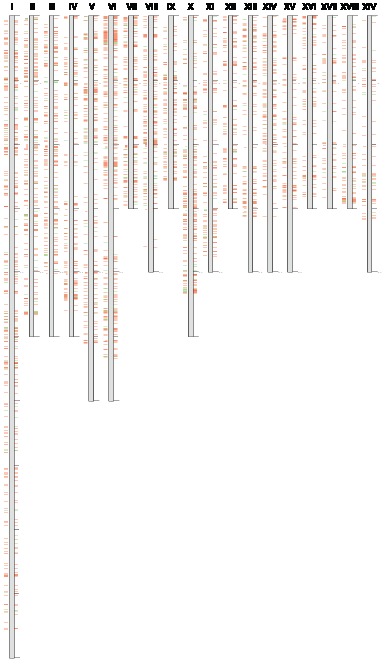
*In silico* mapping of the *P. euphratica* eSSRs onto *P. trichocarpa* pseudo-chromosomes. Gray columns represent 19 pseudo-chromosomes of *P. trichocarpa*. *P. euphratica* eSSRs are arranged on these pseudo-chromosomes, with green lines representing experimentally validated EST-SSRs and red lines indicating EST-SSRs that are not validated.

The selection of evenly spaced markers on linkage groups/chromosomes of interest is typically a crucial issue in molecular breeding, and especially in linkage group construction. Previous genomic and genetic studies have revealed high levels of collinearity for markers (including SSR markers) between *Populus* species [32], [38]. *P. trichocarpa* was the first perennial plant to have its whole genome sequenced. We therefore mapped the newly-developed *P. euphratica* eSSRs onto the scaffolds of *P. trichocarpa*. Using sequence homology and digital mapping, we were able to putatively assign 3,152 eSSRs onto 19 pseudo-chromosomes (major scaffolds) and other un-anchored scaffolds of *P. trichocarpa* (Figure 4 and Table S6). We observed that the eSSRs were unevenly distributed both among and within chromosomes. In addition, eSSRs deserts were also observed on several chromosomes, especially at the end of each scaffold. These phenomenons, which may be due to the differential selection between paralogous chromosomes, are consistent with observations made in previous eSSR-based studies on *Populus* [39].

The 3,839 SSR-containing ESTs were annotated against the GO protein database using Blast2GO [40]. GO assignments were obtaine for 2,202 SSR-containing ESTs. Based on their GO annotations, most of the SSR-containing ESTs were involved in cellular processes (∼21%), metabolism (∼18%), responses to stimuli (∼18%) and biological regulation (17%) (Figure S2 A). More than half of the cellular component GO annotations indicated that the corresponding ESTs were expressed in the cell (Figure S2B), with macromolecular complexes accounting for ∼6% and the membrane-enclosed lumen for ∼2%. The major molecular functions identified from the annotations were related to binding (∼53%) and catalytic activity (∼33%) (Figure S2C).

**Figure 5:**
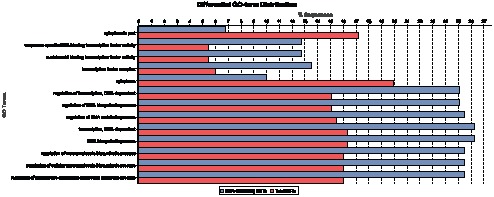
Bar diagram of GO enrichment of SSR-containing ESTs compared to total ESTs. The Y axis represents significantly enriched GO terms and the X axis represents the relative frequency of the GO term.

To determine whether any GO categories were overrepresented among SSR-containing ESTs relative to the case for all ESTs, we performed Fisher's Exact Test (FDR cutoff of p<0.05) using Blast2GO (Figure 5). Some categories were indeed substantially overrepresented among the SSR-containing ESTs, including transcription, regulation of transcription, transcription complexes, RNA biosynthesis and macromolecular biosynthesis (Figure 5). The most heavily overrepresented GO term in the SSR-containing ESTs was sequence-specific DNA binding transcription factor activity (Figure 5), which was typically associated with genes involved in the transcription process. Similar results were obtained in previous studies on *Quercus mongolica* [41] and *Cryptomeria japonica* [30]. However, no GO category was overrepresented among SSR-containing ESTs by more than a factor of 2.5 relative to its representation among all ESTs.

### A web database for *P. euphratica* eSSR markers

To facilitate wider uses of the new *P. euphratica* markers, we constructed a web-based, downloadable and searchable database for *P. euphratica* eSSR markers that also contains transferability data for some of the validated polymorphic markers in nine other *Populus* species. The database can be accessed freely via a web interface (http://202.205.131.253:8080/poplar/resources/static_page/index.html). In addition, we have created a graphical tool that is accessible on the homepage and displays 19 *P. trichocarpa* pseudo-chromosomes (Figure 4) showing the positions of the eSSR markers. This tool enables users to select locations of interest and directly obtain information on the relevant eSSRs. It is possible to search for EST sequences, primer information, and specific annotations in several ways, including searches based on ID, location (CDS or UTR), SSR motif, scaffold, etc.

## Discussion

### Frequency, distribution and location of eSSRs of *P. euphratica*

Due to their co-dominance and high level of polymorphism, SSR markers are the most popular markers in studies on population genetics, QTL mapping, and related areas. EST sequences are the main resources from which new SSR markers are developed [42]. The increasing availability of transcriptome data produced using next generation sequencing methods has greatly enhanced the scope for developing markers in this way. However, both short reads and redundancy are common in next generation data, which can be problematic because only non-redundant ESTs can be used to generate unique SSR markers.

In this study, we identified 4,202 eSSRs from 94,090 non-redundant EST sequences. It thus seems that eSSRs account for around 4% of the transcriptome in *P. euphratica*. To compare the SSR frequency in *P. euphratica* with that in other plants, we used the same software (SSRLocator [43]) and same cut-off as were previously used by Victoria *et al*. [44], focusing on SSR lengths of >20 bp. In our dataset, 2,445 SSRs satisfied these criteria, corresponding to a frequency of around 2.6%. This is comparable to the situation in *Chlamydomonas reinhardtii* (about 2.4%) and *Syntrichia ruralis* (about 2.7%). To compare the SSR density of *P. euphratica* with that in other plant species, we used the same criteria as described in Cardle *et al*. [45] with thresholds of 7, 5, 4 and 3 repeats for di-, tri-, tetra-, and pentanucleotides SSRs, respectively. We identified 6,573 SSRs in total with one SSR for roughly every 9.5 kb (1/9.5 kb) of EST sequence data in *P. euphratica*. This density is lower than that in rice (1/3.4 kb), soybean (1/7.4 kb), and maize (1/8.1 kb). However, it is higher than those previously reported for tomato (1/11.1 kb), *Arabidopsis* (1/13.8 kb), poplar (1/14.0 kb) and cotton (1/20.0 kb). Although the SSR density in *P. euphratica* determined in this work was greater than that reported for poplar by Cardle *et al*., the two results are similar in magnitude. The difference may be due to the relatively small number of EST sequences (1,880 kb) examined by Cardle *et al*. compared to the dataset used in this work (62,650 kb).

Previous studies have identified differences in the abundance of different motifs in eSSRs [46]. In general, trinucleotides repeats are the most common, followed by either di- or tetranucleotides repeats [42]. As in other higher plants, trinucleotides repeats are the most common SSR motifs in *P. euphratica*, accounting for 37% of all its SSRs. This is consistent with the fact that changes in the number of trinucleotides repeats in a coding region will not cause a frame shift [47]. As expected, an analysis of the eSSR distribution showed that trinucleotides repeats were most commonly found in coding regions. We also found that hexanucleotides repeats were frequently found in coding regions, with a similar ratio to trinucleotides repeats. This is further evidence for the positive correlation between the incidence of SSRs in a coding region and the likelihood that a change in the SSR's repeat number will cause a frame shift. In contrast to trinucleotides repeats, our results showed that dinucleotides repeats are preferentially located in non-coding regions, especially the 5′UTR. Interestingly, we found that most of the eSSRs in non-coding regions were located in 5′UTR. This has also been observed in oaks [24] and rice [48], and was proposed to be due to the regulatory role of the 5′UTR in gene expression.

To further explore eSSR motif bias in *P. euphratica*, we analyzed the abundance of each motif type in detail. AG/CT was the most common motif identified, accounting for nearly 10% of the total pool of eSSRs pool. Similar findings have been reported for several other plants, including *Arabidopsis*, maize, soybean, wheat and rice [46], [49]. This seems to be common in the plant kingdom, suggesting that the AG/CT type of motif may have been under positive selective pressure during eSSR evolution. One possible reason for this phenomenon was suggested by Kantety *et al*. [26] who noted that the codons that incorporate AG/CT i.e. GAG, AGA, UCU and CUC correspond to Arg, Glu, Ala and Leu respectively. Of these, Ala and Leu are relatively common in proteins, occurring at frequencies of 8% and 10%. Although AG/CT was the most common single motif, trinucleotides repeats were the most abundant class of eSSRs among the studied ESTs, and AAG/CTT was the most frequent type of trinucleotides repeat. This is consistent with results for dicots such as oak [24], castor bean [50] and *Arabidopsis* [44]. However, in monocots such as rice, CCG/CGG is the most common trinucleotides repeat [46].This difference has been attributed to the high GC content of grass genomes in several studies [49]. In this work, we did not find any GC-containing dinucleotides repeats and only 76 of the 1,573 trinucleotides eSSRs contained GC. This probably reflects the relatively low GC content of dicot genes [49].

### New eSSR markers

There have been several publications describing the development of SSR markers in the genus *Populus* [32], [51], [52]. However, to our knowledge, no more than 12 gSSR markers have been developed for *P. euphratica*. As such, there is a great need to increase the number of available markers for this unique desert tree. Based on the high transferability of SSRs in *Populus* (for example, the transferability rate of markers from *P. trichocarpa* to *P. euphratica* is around 70% [32]), we initially tested over 499 primers that were randomly selected from the OAK RIDGE National Laboratory (http://www.ornl.gov/sci/ipgc/ssr_resource.htm) database. However, less than 20% of the selected primers could be successfully amplified. This could be because EST-derived SSRs, which derived from the transcribed DNA regions, are expected to be more highly conserved and should thus have a higher transferability rate across species than gSSR markers.

Validating SSR primers is important in the early stages of the development process [17]. We have found that the genotyping of SSRs is often laborious and time consuming when using traditional PAGE analysis and so we instead used M13 technology with fluorescent tagging [53]. In addition, pseudomultiplex genotyping based on the use of mixtures of PCR products with different fluorescent labels was also used and found to be an efficient and economical method for identifying SSRs. More than 600 primers were designed and validated in less than one month. 676 of the newly-developed primer pairs were selected at random for amplification to test the reliability of the corresponding eSSR markers in *P. euphratica*, and amplicons were obtained for 575 of them (86%). The instance of unsuccessful amplification may have been due to any of the following factors: 1) The presence of introns in the corresponding ESTs, 2) The presence of SNPs or INDELs in the primers, 3) The tendency of M13 tails to decrease PCR efficiency, leading to a need for additional PCR cycles, 4) Assembly errors in the EST sequences.

### Polymorphism of eSSRs for *P. euphratica*

Only polymorphic markers are useful for population genetic studies at the genomic level. Of the 575 eSSR markers with validated primer pairs, 302 were polymorphic, giving a polymorphism rate of 52.5%. For comparative purposes, it has been reported that 38% of the primer pairs corresponding to a set of eSSR markers successfully amplified a single polymorphic locus each when applied to a reference full-sib pedigree of *Quercus robur* [24]. The polymorphism ratio for *P. euphratica* eSSRs is thus relatively high compared to the values observed in other large-scale polymorphic eSSRs screening studies.

Previous studies have revealed a positive correlation between SSR length and polymorphism rate in plants and animals [54]. We confirmed this observation by comparing the lengths of each type of polymorphic SSR to monomorphic SSRs. Based on studies of dinucleotides, trinucleotides and hexanucleotides repeats, polymorphic eSSRs all have higher number of repeats than monomorphic eSSRs (Figure S3). For all SSR types considered, the repeat number had a significant effect on the rate of polymorphism: the estimated correlation coefficients for this relationship in di-, tri-, and hexanucleotides repeats were 0.091, 0.269 and 0.421, respectively, with corresponding p values of p<0.001, p<0.001and p<0.05. This relationship persisted even when the data for all three SSR types were combined, giving an estimated correlation coefficient of 0.149 (p<0.001).

### A web-based database of eSSRs resource for *P. euphratica*

There are quite few plant SSR databases. The Triticeae eSSR coordination (http://wheat.pw.usda.gov/ITMI/EST-SSR/) provides a collection of 2,463 nonredundant "Uni-EST-SSR" contigs from wheat, barley, rice, maize and sorghum, together with their consensus sequences [55]. The Simple Sequence Repeat Database (http://intranet.icrisat.org/gt1/ssr/ssrdatabase.html) provides eSSRs from sorghum, soybean, medicago, lotus, rice and maize and can be queried based on motif, minimum length, maximum length, and match type [56]. This database also provides cross-species eSSR clusters. The tomato SBM & Marker Database (http://marker.kazusa.or.jp/Tomato/) provides eSSRs and gSSRs for the tomato, but only with the corresponding primer sequences [57]. The CMD (http://www.cottonmarker.org/) incorporates all publicly available SSRs and related information [28]. The *P. trichocarpa* genome has been released and its SSRs have been developed and deposited in the archives of The International *Populus* Genome Consortium (http://www.ornl.gov/sci/ipgc/ssr_resource.htm). However, these data can only be obtained in an EXCEL file that provides limited information, such as primer sequences, amplification status, motif type etc. [32], [51], [52] Our Euphrates Poplar EST-SSR DB integrates both general information regarding its eSSRs (such as the associated EST sequences, primers that have been designed against them, motif type information, polymorphic content, and transferability) and has several other useful features. Notably, it provides the location of each SSR (CDS or UTR) via a graphical interface that shows their relative position on the 19 *P. trichocarpa* pseudo-chromosomes and provides links to more detailed information on SSRs of interest. It also displays annotations for SSR-containing ESTs and makes all of the available information on each eSSR searchable and interlinked.

In conclusion, we have developed and characterized the first large-scale set of eSSRs for *P. euphratica* by analyzing public transcriptome. More than 300 polymorphic eSSR markers have been screened and validated, and the resulting data have been integrated into a user-friendly web-based database. This information will have many potential applications in studies on *Populus* in fields such as population genetics, comparative genomics, linkage mapping, QTL, and marker-assisted breeding.

## Materials and Methods

### Plant material and DNA isolation

Three populations were sampled from a 4×10^4^ hectare poplar forest (Latitude 41.0325°–41.0825°, Longitude 86.1181°-86.4693° and Altitude: 884 m) located along the Tarim River in western China (Yuli County, Xinjiang) in Xinjiang province, China. Six, 12 or 24 individuals were selected at random from each of the three populations. Leaves from each individual were collected and dried over silica gel. The dried leaves were placed in a 2 mL tube, two φ3 mm steel beads were added, and the leaves were ground in a shaker (Mini-Beadbeater, Biospec USA). DNA was extracted using the CTAB method with some modifications [58]. DNA concentrations were measured using a Nano Drop 2000 spectrophotometer (Thermo, USA) and the extracted DNA was transferred to 96 well plates at an adjusted concentration of 10-15 ng/ µL.

### EST sources and eSSR analysis

ESTs from *P. euphratica* were obtained from the NCBI EST database and the transcriptome data reported by Qiu *et al*. [34]. PolyA/T sequences at the 5′ and 3′ ends of the ESTs were removed using EST-trimmer (http://pgrc.ipk-gatersleben.de/misa/download/est_trimmer.pl). ESTs that were below 100 bp in length were not included in the analyses. Vector, chloroplast and mitochondrial sequences were removed from the data set using SeqClean (http://compbio.dfci.harvard.edu/tgi/software/). Redundant ESTs were cleared with CD-HIT [35] using a 90% sequence similarity threshold. Non-redundant ESTs were then searched for SSRs using MIcroSAtellite identification tool (MISA) [36]. We searched for di- (repeat count > = 10), tri- (> = 6), tetra- (> = 5), penta- (> = 4), hexa- (> = 3) nucleotides, with an interruption (max_difference_for_2_SSRs) of 100 bp.

### Mapping and annotation of eSSRs

We used a series of in-house perl scripts to map *P. euphratica* eSSRs onto the *P. trichocarpa* genome. First, the BLASTN program (http://blast.ncbi.nlm.nih.gov/) was used to locate the ESTs on the *P. trichocarpa* genome. Second, BLAST results and information on the locations of the SSRs were combined to infer SSR locations in the *P. trichocarpa* genome. At this stage, only blast results with an identity score of >0.8 were used. Third, we aligned the SSRs against the corresponding sequences in the *P. trichocarpa* genome using MUSCLE (http://www.ebi.ac.uk/Tools/msa/muscle/) with the default criteria. Finally, based on the SSRs' locations in the *P. trichocarpa* genome and gene annotation data for the *P. trichocarpa* genome, the location of each SSR with respect to the nearest gene (UTR or CDS) was evaluated and the mapping results were recorded in an EXCEL document (Table S6).

Functional annotations were assigned to ESTs and eSSRs using Blast2GO [40]. In brief, BLAST was used to find sequences that were similar to each EST by searching against the NCBI non-redundant protein database with an e-value cutoff of 1e-3. Then GO terms associated with each blast hit were extracted and the resulting GO annotations were returned. The associated cellular component, biological process, and molecular function were identified for each EST. Fisher's Exact Test was performed in Blast2GO to identify overrepresented GO categories in SSR–containing ESTs compared with total unigenes using an FDR cutoff of <0.05.

### Design of PCR primers and SSR genotyping

Primers for eSSR amplification were designed in batches using the PRIMER3 program [59] in conjunction with MISA [36] and in-house perl scripts. Information on each primer pair, including their sequences, positions, and *Tm* values, are presented in Table S3.

We selected 676 primer pairs at random from the primer database to assess their validity. For primary primer screening, PCR reactions were performed with a 5′ M13-tailed forward primer and reverse primer pair using DNA from four randomly selected *P. euphratica* individuals sampled in Xinjiang Province, China. Each 25 µL polymerase chain reaction (PCR) mixture contained 1x Taq buffer, 0.2 mM dNTPs, 10-20 ng template DNA, 1.6 pmol of the 5′ M13-tailed forward primers and the reverse primer each, and 1 U *Taq* polymerase (BioMed). The PCR conditions were as follows: 94°C for 5 min; then 30 cycles of 94°C for 30 s, annealing at 56°C for 45 s, elongating at 72°C for 45 s, and a final extension at 72°C for 10 min. 5 µL of the PCR products were separated on 2% agarose gels and stained with ethidium bromide to check for successful amplification. After primary primer screening, SSR genotyping was then performed with primer pairs that yielded amplicons. At this stage, DNA from six to 12 or 24 individuals from three to four locations across the distribution of *P. euphratica* was used to investigate the polymorphism of the new eSSR markers using a rapid and inexpensive method based on the M13-tail technique [53]. The detailed M13-tail technique used in this study was as follows. Three primers were synthesized for each genotyping run: a 5′ M13-tailed forward primer, a reverse primer, and a fluorescently-labeled M13 primer. The M13 primers were labeled with FAM, ROX, HEX, and TAMRA (Sangon) respectively. Each 10 µL polymerase chain reaction (PCR) contained 1x Taq buffer, 0.2 mM dNTPs, 10-20 ng template DNA, 1.6 pmol of the reverse and fluorescently-labeled M13 primers each, 0.4 pmol of the forward primer, and 1 U *Taq* polymerase (BioMed). The PCR conditions were as follows: 94°C for 5 min; then 30 cycles of 94°C for 30 s, annealing at 56°C for 45 s, elongating at 72°C for 45 s, followed by 8 cycles of 94°C for 30 s, annealing at 53°C for 45 s, elongating at 72°C for 45 s, and a final extension at 72°C for 10 min. Subsequently, 0.5 µL of each PCR product with the four different fluorescent dyes were mixed and added to 10 µL formamide and 0.5 µL LIZ standard (ABI) and run on an ABI 3730 Prism genetic Analyzer. Raw data were analyzed using GeneMarker Version 1.75 (Softgenetics, USA).

### Evaluation of eSSR polymorphism and genetic diversity analysis

Genetic diversity was calculated at each locus for allelic PIC using the PIC calculator (http://www.liv.ac.uk/~kempsj/pic.html) based on allelic frequencies among all the genotypes analyzed. PIC values for each SSR were estimated by determining the frequency of alleles per locus using the formula: PIC = 1-Σ(*P*_i_)^2^, where *P*_i_ is the relative frequency of the *i*th allele of the SSR loci. The expected heterozygosity (*H*_E_) and number of alleles (*N*_A_) were estimated for six to 12 or 24 individuals using GENALEX 6 [60]. The effect of repeat number on the polymorphism was tested using a logistic regression model created with v 2.6.2 of the R software package (R development core team 2008).

### Transferability of polymorphic eSSRs to other *Populus* species

The transferability of the new polymorphic eSSRs was evaluated in nine other species (three endemic in China) belonging to five sections of the *Populus* genus. Three species were selected from the Tacamahaca Spach section, two each from the Aigeiros Duby and Leucoides Spach sections, and one each from the Leucoides Duby and Turanga Bge sections. PCR reactions were performed using primer pairs corresponding to the polymorphic eSSRs in one to four individuals of each *Populus* species. If a primer pair yielded successful amplification, the corresponding eSSR was scored as being transferable.

### Phylogenetic tree construction

To determine whether there was any correlation between the observed rates of transferability and the phylogeny of the *Populus* genus, we constructed a Neighbour joining tree by assigning successful amplifications a value of ‘1’ and amplification failures a value of ‘0’. The final Neighbour joining tree for the 10 species was constructed using over 300 information sites by MEGA5 [61] with 1000 bootstraps.

### Construction of a web-based *P. euphratica* eSSR database

In order to share our data and analytical results on *P. euphratica* eSSRs, including ESTs, SSRs, the relative positions of each SSR on the 19 *P. trichocarpa* pseudo-chromosomes, primer sequences, polymorphisms, and transferability, we integrated the data from each step of our investigation into a web-based *P. euphratica* eSSR database based on open-source software (Apache, PHP, and MySQL). Our *P. euphratica* eSSR database has a user-friendly interface and is freely available at the following website: http://202.205.131.253:8080/poplar/resources/static_page/index.html.

## Supporting Information

Figure S1 **Frequency distribution of different size of motifs in EST-SSRs of *Populus euphratica*.**
(TIF)

Figure S2 **GO ontology of SSR-containing ESTs was categorized by biological process (A), cell component (B) and molecular function (C).**
(TIF)

Figure S3 **Correlation between PIC and motif size of EST-SSRs of *Populus euphratica*.**
(TIF)

Table S1 **Occurrence of 4202 SSRs identified in a set of 94,090 non-redundant *Populus euphratica* ESTs.**
(XLS)

Table S2 **Frequency of different motif types.**
(XLS)

Table S3 **Primer information for all the 3,069 EST-SSRs.**
(XLS)

Table S4 **Characteristics of the 673 EST-SSRs tested in six, twelve or 24 individuals of *Populus euphratica*.**
(XLS)

Table S5 **Transferability of all the polymorphic EST-SSRs in other nine Poplar species.**
(XLS)

Table S6 **Location of the *Populus euphratica* EST-SSRs on the genome of *Populus trichocarpa***.
(XLS)
