# Supplementary figures and images for: Exploiting the Transcriptome of Euphrates Poplar, Populus euphratica (Salicaceae) to Develop and Characterize New EST-SSR Markers and Construct an EST-SSR Database
Source: PLoS One. 2013 Apr 11;8(4):e61337. doi: 10.1371/journal.pone.0061337 (PMC3623821; doi:10.1371/journal.pone.0061337)

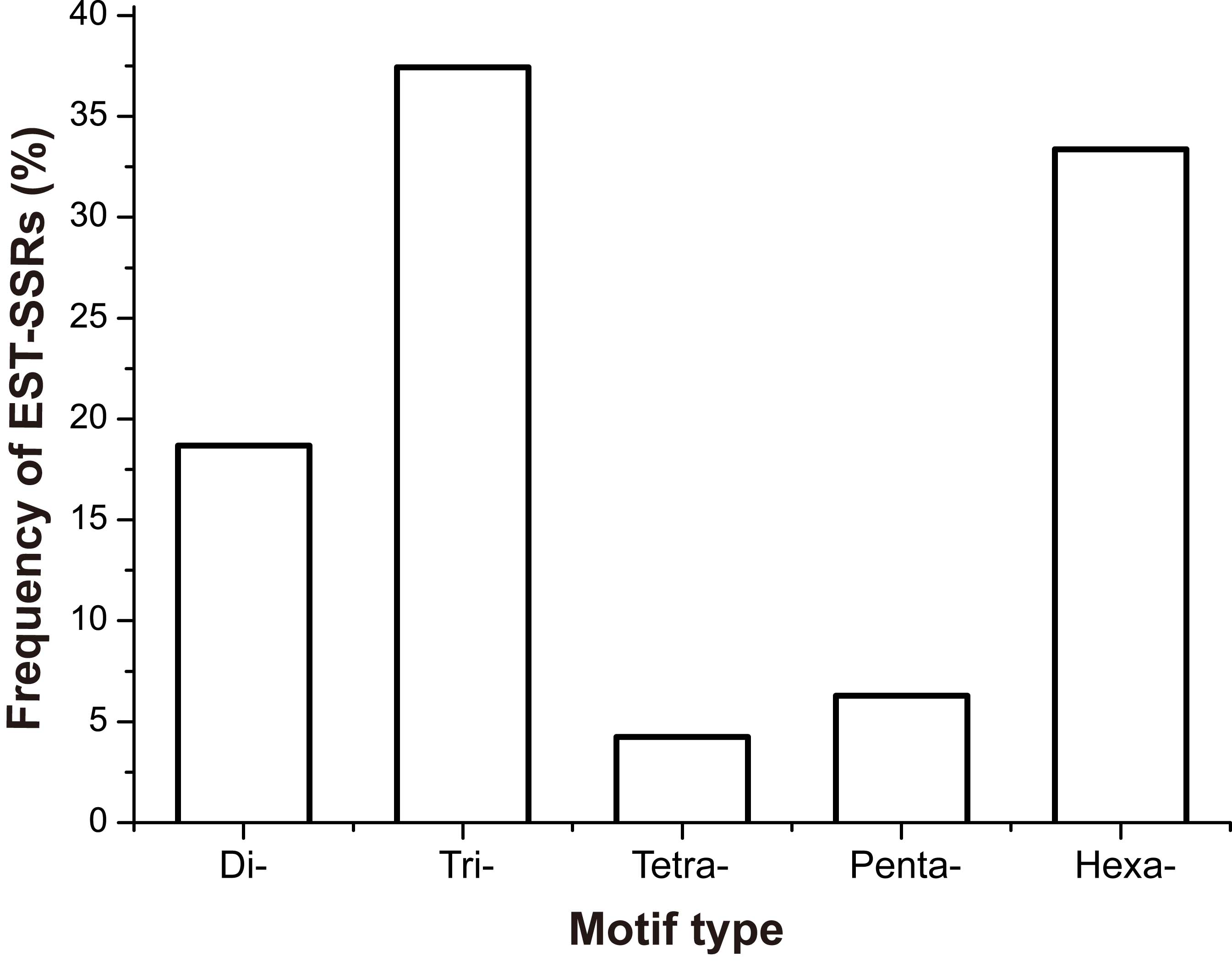

Supplement: Figure S1 — Frequency distribution of different size of motifs in EST-SSRs of Populus euphratica. (TIF) [file pone.0061337.s001.tif]

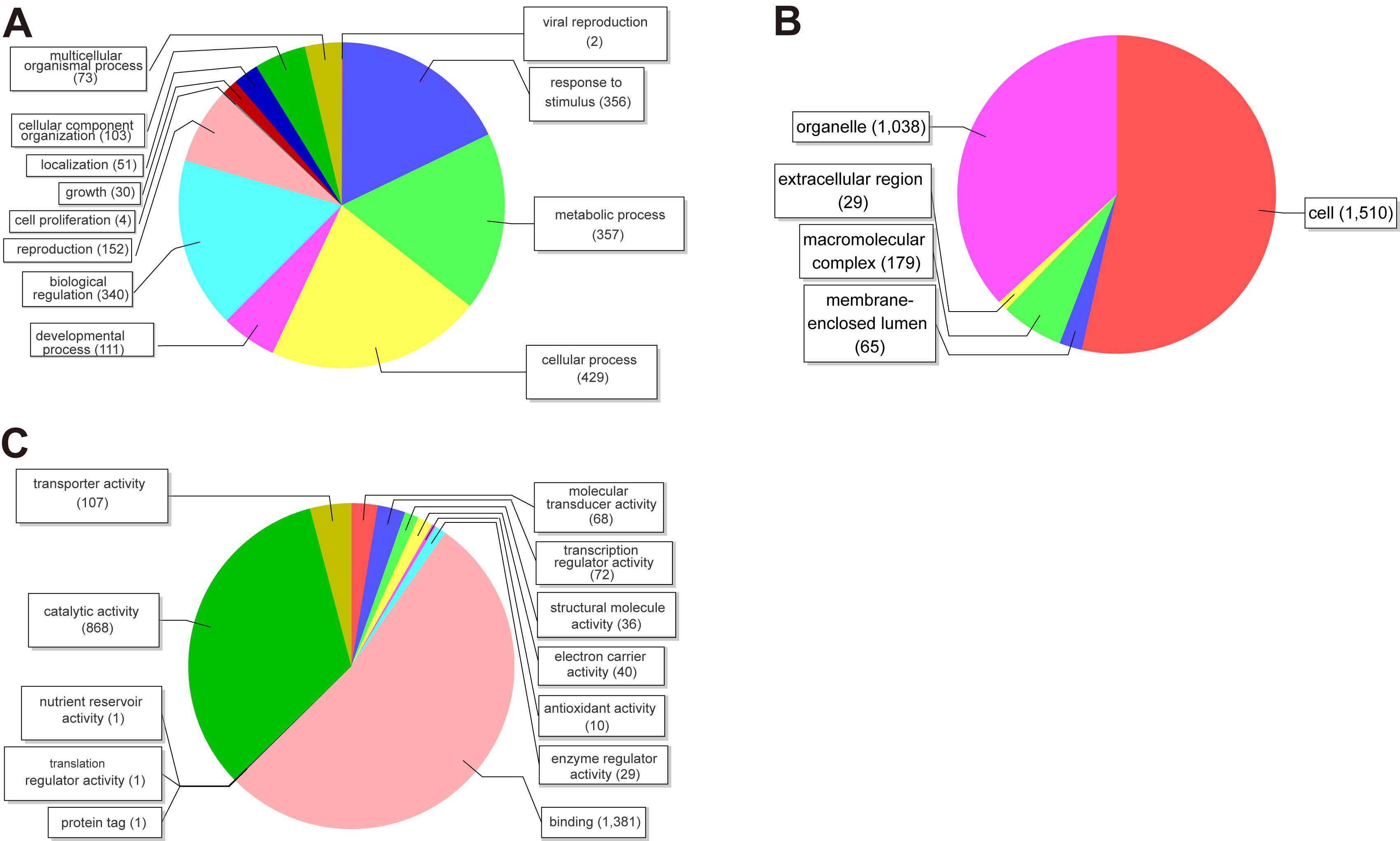

Supplement: Figure S2 — GO ontology of SSR-containing ESTs was categorized by biological process (A), cell component (B) and molecular function (C). (TIF) [file pone.0061337.s002.tif]

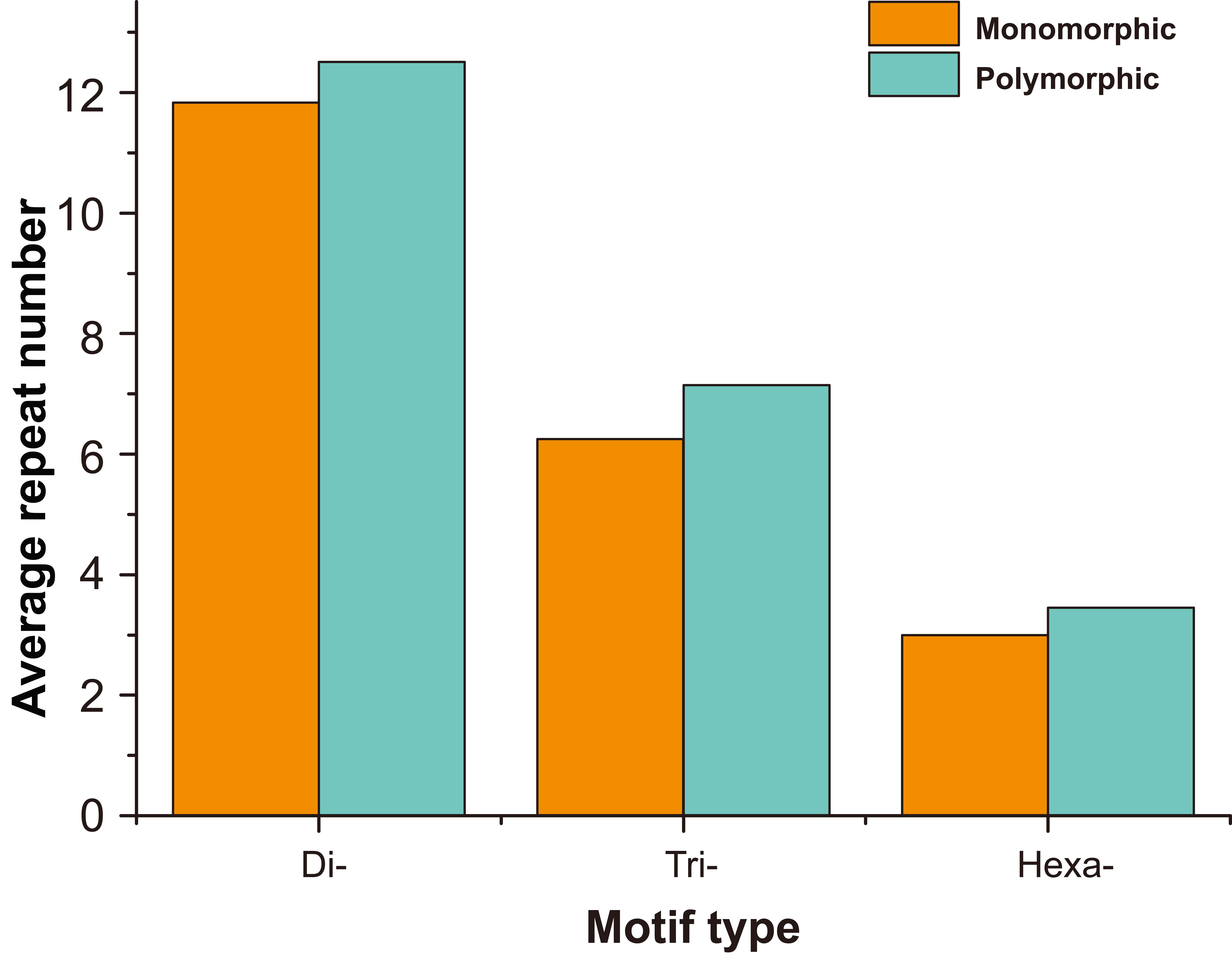

Supplement: Figure S3 — Correlation between PIC and motif size of EST-SSRs of Populus euphratica. (TIF) [file pone.0061337.s003.tif]
